# Exploring sub-species variation in food microbiomes: a roadmap to reveal hidden diversity and functional potential

**DOI:** 10.1128/aem.00524-25

**Published:** 2025-04-30

**Authors:** Lena Flörl, Annina Meyer, Nicholas A. Bokulich

**Affiliations:** 1 Department of Health Sciences and Technology, ETH Zurich 311632 , Zurich, Switzerland; Michigan State University, East Lansing, Michigan, USA

**Keywords:** microbiome, metagenomics, food systems, strain profiling

## Abstract

Within-species diversity of microorganisms in food systems significantly shapes community function. While next-generation sequencing (NGS) methods have advanced our understanding of microbiomes at the community level, it is essential to recognize the importance of within-species variation for understanding and predicting the functional activities of these communities. This review highlights the substantial variation observed among microbial species in food systems and its implications for their functionality. We discuss a selection of key species in fermented foods and food systems, highlighting examples of strain-level variation and its influence on quality and safety. We present a comprehensive roadmap of methodologies aimed at uncovering this often overlooked underlying diversity. Technologies like long-read marker-gene or shotgun metagenome sequencing offer enhanced resolution of microbial communities and insights into the functional potential of individual strains and should be integrated with techniques such as metabolomics, metatranscriptomics, and metaproteomics to link strain-level microbial community structure to functional activities. Furthermore, the interactions between viruses and microbes that contribute to strain diversity and community stability are also critical to consider. This article highlights existing research and emphasizes the importance of incorporating within-species diversity in microbial community studies to harness their full potential, advance fundamental science, and foster innovation.

## FOOD SYSTEMS ARE AN IDEAL MODEL TO SHOWCASE AND INVESTIGATE WITHIN-SPECIES VARIATION

Different strains belonging to the same species can vary drastically in their functional potential, resistances, and pathogenicity. In microbial communities, within-species variations can contribute to the distinct function and properties of entire microbiomes. In food systems, such within-species variation therefore conveys unique characteristics and influences the quality and safety of a product (1–5). The resulting properties can be unique to an extent that it allows for the authentication of protected designation of origin and protected geographical indication products by tracking distinct associated strains (6, 7). Furthermore, strain tracking plays a crucial role in monitoring and mitigating disease outbreaks and contamination in food production facilities (8). Hence, as these effects are particularly easily observable, literally tastable, fermented foods and food systems are an ideal model for studying within-species diversity. Additionally, their wide availability, greater number of culturable species, and relatively simpler microbial consortia enhance their suitability as study systems (9). But how can these complex microbial communities and their variation within be efficiently and comprehensively studied? Here, we give examples for the effect of within-species diversity in food systems, review state-of-the-art methodologies, and showcase their applications.

## THE EMERGENCE OF WITHIN-SPECIES DIVERSITY AND ITS IMPLICATIONS

**Fig 1:**
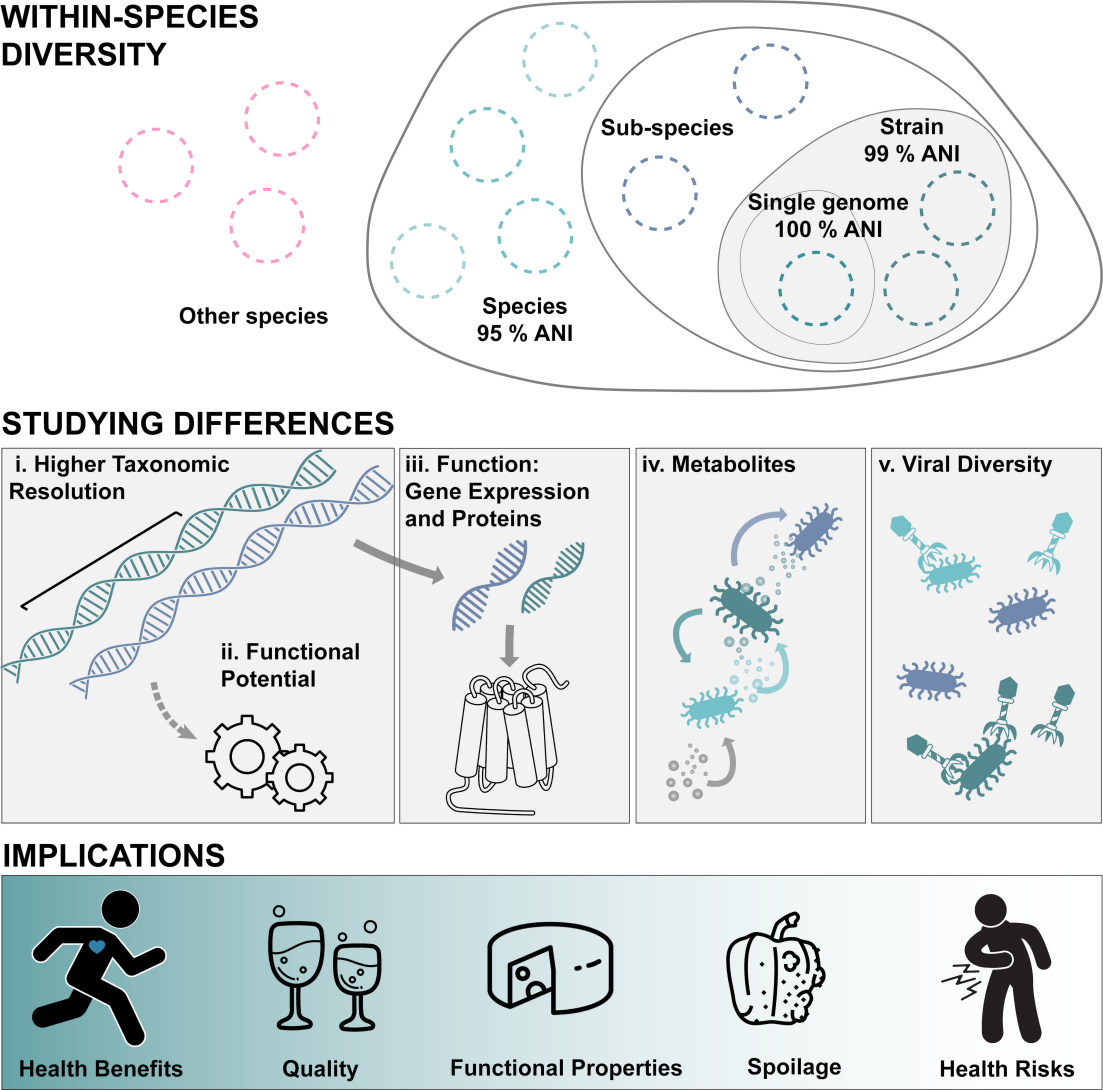
Summarizes the contents of this article. WITHIN-SPECIES DIVERSITY: The term “strain” lacks a definitive definition and varies by field, but is commonly understood as a phenotypically and/or genotypically similar subset of a species (ANI in the core genome >99%). A species can comprise multiple sub-species, each sub-species may contain several strains, and a strain may consist of multiple distinct genomes (for a more detailed review on this complex issue, see Van Rossum et al. [14]). STUDYING DIFFERENCES: Different strains can have distinct properties, and here, we outline different technologies to study these. (i) Covering larger regions of the genome with long-read sequencing or whole metagenome sequencing provides higher resolution of microbial communities. (ii) This metagenomic data can also reveal the functional potential of individual strains or communities. (iii) Methods such as metatranscriptomics and metaproteomics allow for a deeper exploration of microbial functions and the distinct effects of different strains. (iv) Metabolomics, the analysis of the chemical environment surrounding and modulated by microbes, offers insights into strain development and functionality within ecological systems. (v) Additionally, understanding the interactions between viruses and microbes is crucial, as these relationships play a key role in shaping strain diversity and maintaining community stability. IMPLICATIONS: These diverse functionalities and phenotypes have distinct implications for food production.

Microbial communities in food systems naturally assemble from a species pool originating from raw materials, production facilities, and likely even the food producers themselves (10, 11), with environmental conditions acting as a selective pressure (12). Genetic diversification selects for the emergence of novel strains with novel functions, whose growth is enhanced, reduced, or even inhibited by biotic and abiotic factors encountered in the surrounding environment (12). Additionally, bacteria and archaea have an open gene pool and exchange genetic elements through horizontal gene transfer (HGT), a process that occurs more frequently in related organisms but is even observed across domains (13). The magnitude of this gene flow is determined by the genetic similarity between organisms as well as their natural competence and recombination machinery. Generally, a higher degree of genetic similarity, or relatedness, increases DNA exchange, subsequent recombination, and genomic integration of novel genes or even entire gene clusters (14). Ecological overlap, i.e., the close physical proximity and shared ecological niche with exposure to the same selective conditions, increases the likelihood of exchanging genes (14). Such highly localized, micro-evolutionary changes can lead to a large genomic diversity for single species within the same sample (15). This genetic flexibility complicates the delineation of species and, even more so, strains. Hence, the terminology surrounding microbial classification is often inconsistent, leading to significant confusion (15). Species are typically delineated by having >95% average nucleotide identity (ANI) across the core genome, and further subdivided into distinct strains which are genetically (>99% ANI) and/or phenotypically similar, with the specific definition potentially varying by research field (14, 16) (see Fig. 1). Strains can highly vary in metabolic capabilities, phenotype, or stress response. This ostensible redundancy of strains co-existing in the same system is expected to produce unique characteristics and to increase community stability against viral invasions and environmental fluctuations (17).

## EXAMPLES FOR WITHIN-SPECIES VARIATION IN FOOD SYSTEMS

The described genetic stratification leads to phenotypic and functional variation, which is evident across numerous key species in food systems. First and foremost, *Saccharomyces cerevisiae* is one of the most important species in fermented foods and contains a huge and highly specialized variety of strains (18). This within-species diversity arose from the millennia of co-domestication of the yeast with humans fermenting fruits and cereals. Hence, strains demonstrate significant adaptation to specific nutrient sources and demonstrate genotypic and phenotypic variation between different types of fermentations (e.g., wine, beer, sake, bread), as well as having highly variable stress responses (e.g., to sulfur or ethanol), nitrogen and vitamin acquisition (19). On the other hand, many strains of *S. cerevisiae* act as food spoilage organisms, exhibiting undesirable qualities, for example, in olives or cheese (20), and some strains are opportunistic human pathogens (21) or probiotics in the case of *S. cerevisiae* var. *boulardii,* the most common commercial human probiotic yeast (22).

Similarly, *Penicillium roqueforti* strains exhibit particularly high phenotypic variability (e.g., spore formation, growth rate, salt tolerance) and can strongly vary in the production of secondary metabolites (23). Certain compounds produced by some strains are considered beneficial to human health (e.g., mycophenolic acid and andrastin A) (24), while other strains can produce secondary mycotoxins or other secondary metabolites that can cause serious illness (23). Additionally, proteolytic activities as well as aroma formation vary strongly, and specific strains are therefore kept and propagated through back-slopping for centuries to produce desired characteristics (23). *P. roqueforti* strains form such distinct genetic clusters in different ecological niches, such as in cheese production and food spoilage, that they are no longer capable of genetic recombination (23).

The within-species variability of many other foodborne yeasts and bacteria diverges between beneficial and detrimental. For example, various strains of the yeast *Pichia kudriavzevii* exhibit human pathogenic or probiotic properties (25). The species is highly relevant for the food industry, used to convey functional properties (e.g., shelf-life, texture) or to improve sensory and nutritional qualities, while some strains also exhibit food spoilage potential (26). This within-species variation, which can determine whether *P. kudriavzevii* acts as a beneficial strain in food fermentations or as a spoilage agent, is also evident in species such as *Debaryomyces hansenii* (27), *Hanseniaspora uvarum* (28), *Levilactobacillus brevis* (formerly *Lactobacillus brevis*) (29), and *Penicillium roqueforti* (23).

This list of the implications of within-species variation in food systems could be extended to include *Lactiplantibacillus plantarum* (formerly *Lactobacillus plantarum*), known for its notably large genome, exceptional metabolic adaptability, and extensive ecological range (30). Similarly, *Hanseniaspora uvarum* demonstrates overall phenotypic uniformity yet harbors substantial genetic diversity that clusters distinctly across spatial and temporal scales (28). Such within-species variations, with critical functional relevance in food systems, have also been documented for various other fungi and bacteria (31–41).

The relative importance of strain-level diversity likely varies across fermented foods and food systems. In some cases, functional variation is primarily species-driven, as with *Saccharomyces cerevisiae* ensuring alcohol production in wine fermentation. In other contexts, any strain variations are critical, such as wild *S. cerevisiae* strains contaminating beer fermentations and causing flaws like over-attenuation, haze, or off-flavors (42). We speculate that systems prone to overgrowth by cross-contaminants or wild strains, like non-starter lactic acid bacteria (LAB) in cheese (43), may depend more heavily on strain-level variation and therefore would require stricter strain monitoring and management to maintain desired fermentation outcomes. While within-species variation undoubtedly contributes to the unique properties of a fermented product and food safety, the extent to which it interacts with environmental factors and interspecies dynamics remains difficult to disentangle. Comparative studies evaluating the relative importance of strain-level variation across food systems are still notably lacking.

## CURRENT APPROACHES TO STUDY MICROBIAL DIVERSITY: DIVERGENCE BETWEEN CULTIVATION-BASED AND SEQUENCING-BASED METHODS

Techniques to isolate and grow microorganisms in solid or liquid media laid the foundation of the field of microbiology in the mid-19th century, and to this day are key routine applications for enumerating viable organisms or studying their phenotype and behavior, e.g., metabolism (44). Cultivation therefore remains highly relevant to discover new strains (4) and investigate their functionality in response to abiotic factors or community interactions (2, 9). Notably, the growth of microorganisms depends on chosen cultivation conditions and is inherently biased by the media type, oxygen availability, incubation time, and temperature, etc. Due to this selectivity, the bottom-up approach of cultivation-based methods lacks information on the composition and interactions within the actual microbial community (45). Axenic cultivation often poorly represents the natural environments in which microorganisms are found, and hence skews assessments of microbial phenotypes, e.g., growth or metabolism (46). Studies using both culture-dependent and -independent approaches have shown that cultivation does not recover the actual microbial diversity and often overlooks even dominant taxa or fails to detect significant differences between production sites (47, 48). Culture-independent approaches utilizing next-generation sequencing (NGS) technology enable a top-down approach to profile microbial communities. NGS is based on massive parallelization of millions of sequencing reactions occurring simultaneously (49). This facilitates rapid and deep sequencing of whole genomes of isolated strains, but also allows for the sequencing of all present DNA in a sample (shotgun metagenomics) or profiling microbial communities with representative marker genes, e.g., 16S SSU rRNA for bacteria and archaea, or the internal transcribed spacer domain for fungi (49). The relative inexpensiveness, availability of robust protocols, as well as broad establishment in the field makes marker-gene NGS using Illumina sequencing-by-synthesis technology the most commonly used tool to profile microbial communities in food systems (50). However, the actual taxonomic resolution depends on the marker gene and primer choice, among other factors. The most commonly used bacterial primers cover only one or two hypervariable regions of the 16S rRNA gene and are not sufficient to reliably resolve genetic differences within a species, and even the full-length 16S rRNA gene is insufficient to fully resolve some species (4, 51). Hence, focusing exclusively on the broader community level risks overlooking the importance of within-species variation. To prevent a divergence between these two approaches that may create isolated subfields, we suggest bridging this emerging gap between cultivation-dependent and -independent approaches for studying microbial diversity in fermented foods.

## A ROADMAP TO STUDY MICROBIAL DIVERSITY CONSIDERING WITHIN-SPECIES VARIATION IN FOOD SYSTEMS

### Technologies that provide higher taxonomic resolution are crucial for comparing microbial communities in similar ecological niches or tracking microbial dispersal

The genetic differences between sub-species variants within similar ecological niches, which have been exposed to the same selective pressures, can be minimal. Thus, relying solely on Illumina marker-gene sequencing, which at the time of writing provides a maximum read length of 2 × 300 bases (52), may be insufficient to detect subtle differences when comparing microbiomes in closely related ecological niches (Table 1). Technologies able to retrieve longer-read lengths, like Oxford Nanopore sequencing or single-molecule real-time sequencing used in PacBio devices, can cover a larger section of a marker gene and accordingly increase resolution, provided that appropriate markers are targeted. For comparison, long-read sequencing technologies offer read lengths up to 20 kilobases for PacBio (53) and up to megabase-scale long reads for Nanopore (54). While long-read marker-gene sequencing can thereby reliably resolve intragenomic variation, e.g., of 16S rRNA alleles within a species, current taxonomic assignment methods often lack the resolution to distinguish sub-species variants due to the absence of strain-level annotated reference databases (55). Furthermore, the issue of strains potentially containing multiple, distinct rRNA operon copies remains unresolved by existing approaches (55) (see Table 1).

**TABLE 1:** Different DNA sequencing techniques provide varying degrees of resolution, with higher resolution typically associated with increased costs^*a*^

Method	Resolution	Advantages^*b*^	Caveats^*c*^	Application examples	Cost
Illumina marker-gene sequencing	Species, sub-species variants (14)	+ Robust + Well-established protocols + Extensive databases + Robust bioinformatics tools	Resolution strongly depends on – choice of marker gene – primers – bioinformatic tools (56)	Microbial community profiling Monitoring population shifts Initial screening and basic quality control	$
Long-read marker-gene sequencing	Species, strain resolution possible (55)	+ Increased resolution + Improved phylogenetics (57)	Still difficulties in distinguishing strains: – marker genes too conserved – primer/target selection – multiple marker-gene copies – no databases with annotated variants (58)	Pathogen detection and tracking (58) Microbial fingerprinting: authentication (7)	$$
Metagenome sequencing	Strain level (59)	+ Discovery of novel strains + Assessing functional potential	Resolution strongly depends on – sequencing depth – run quality – computational resources (60)	Identifying novel probiotics Revealing strains and associated mechanisms of food spoilage (5, 8)	$$$
Single-cell DNA sequencing	Single amplified genomes (61)	+ Highest resolution of single-microbe genomic information (61)	– not widely applied in microbiome research yet	Spread of antibiotic resistances Studying genetic variations in fermented foods	$$$$

^*a*^ It is crucial to distinguish between technical resolution, which relates to the sequencing method itself, and taxonomic resolution, which depends heavily on the quality and comprehensiveness of reference databases. To further clarify the hierarchy of microbial classification, a species can include multiple sub-species, each sub-species may comprise several strains, and a single strain can consist of multiple distinct genomes (14).

^*b*^ +, advantages.

^*c*^ -, drawbacks.

Therefore, a further step up in distinguishing within-species variants is metagenomic-assembled genomes (MAGs), which are reconstructed from shotgun metagenome sequencing data. Since shotgun sequencing gives insight into the entire genomic information content of a sample, it also allows for entirely new strain discovery, provided that sequencing was performed at an adequate depth to enable assembly of MAGs or use of read-based methods for strain detection (62). This is further aided by using long-read sequencing in metagenomics, which improves the identification of structural variations and thereby drastically enhances taxonomic resolution (63). Yet, as long-read marker-gene sequencing and shotgun metagenome sequencing (in sufficient depth to assemble MAGs) are still significantly more expensive than Illumina-based marker-gene sequencing (i.e., roughly an order-of-magnitude difference at the time of writing), these technologies are sometimes applied only to a subset of samples. Notably, shotgun metagenome data additionally require significantly more computational power and resources for analysis, compared to marker-gene amplicon sequence data. Sequencing quality and depth also limit how many organisms can be distinguished down to a sub-species level, which is commonly only possible for dominant species (59) (see Table 1). Despite these barriers, metagenomics analysis is considered to have huge potential in monitoring and mitigating contaminations and outbreaks in food production facilities by enabling tracing and investigating the persistence of distinct strains (8).

Ultimately, single-cell DNA sequencing (scDNA-seq) enables the resolution of individual genomes from single organisms in complex microbial communities through single-amplified genomes (61). Despite remaining challenges and barriers such as variable lysis efficiency, uneven genome amplification, high computational demands, elevated costs, as well as relatively low throughput, scDNA-seq methods hold significant potential for advancing our understanding of within-species microbial diversity (64, 65) (see Table 1). However, these techniques have yet to be broadly applied in microbiome research or specifically to food systems data sets, most likely due to significant barriers imposed by cost, accessibility, and technical challenges.

Overall, higher resolution sequencing technologies can more effectively uncover the within-species variability in similar ecosystems and are particularly valuable for applications such as tracking strain dispersal in food processing environments (66) or identifying pathogenic strains in food safety (67).

### Shotgun metagenomics can elucidate the functional potential of strains or communities, guide isolation, reveal associations between metabolic pathways, and find relevant gene clusters

Assembled MAGs from shotgun metagenomics data also offer valuable insights into the functional potential of individual strains and entire microbial communities. From this functional potential, certain phenotypes, such as estimated growth conditions including optimal temperature, oxygen tolerance, salinity, and pH, can be predicted (68). The use of NGS data to guide subsequent cultivation is termed culturomics (69) and has enabled the isolation of previously uncultured microorganisms. For instance, Landis et al. (70) utilized shotgun metagenomic sequencing data to guide the isolation of key strains, revealing that within-species variation among Kombucha strains, despite over 99% genomic similarity, significantly influenced biofilm formation and metabolite production (70).

Additionally, the functional annotations can elucidate metabolic pathways, antimicrobial resistance genes, potentially health-associated gene clusters (PHAGCs), or other biosynthetic gene clusters. For example, Leech et al. (71) applied shotgun sequencing and MAG assembly to 58 different fermented foods, identifying 10 putatively novel strains and assessing antimicrobial potential, such as bacteriocin production and other PHAGCs (71). Similarly, shotgun sequencing has been applied to assess how packaging conditions of beef select for distinct strains with spoilage potential (72) or to expand our knowledge of how food microbiomes link to the human microbiome and potentially impact health (73).

Correlating characteristics, e.g., volatile organic compound (VOC) production, with the predicted functional potential of bacterial and yeast strains has further been employed to screen dynamics and impact on cheese quality (74). Additionally, it has been used to reconstruct metabolic pathways of flavor compounds in distinct lactic acid bacteria strains found in traditional Mexican Cotija cheese (75). Moreover, improved accuracy of functional annotations also permits the construction of genome-scale metabolic models (GEMs). While currently limited to a few species, multiple GEMs can be integrated to simulate intermicrobial interactions that would be difficult to investigate *in vivo*, as strain-specific differences may confer differential community behaviors (76).

### Exploring strain-specific functional differences and responses to specific conditions using metatranscriptomics and metaproteomics

While metagenomics reveals a community’s functional potential, metatranscriptomics examines active gene expression and functional response to changed conditions (77). This approach allows us to understand microbial community responses to particular conditions and gain deeper insights into the functional differences between species and strains. Studies on kimchi (78) and cheese rind microbiomes (79) have shown species- and strain-specific gene expression impacting key metabolic pathways, e.g., carbohydrate metabolism and iron utilization. In both studies, assembled MAGs from shotgun metagenomics were used as reference genomes for mRNA transcript mapping and identification of upregulated or downregulated genes as well as entire metabolic pathways. Apart from comparing gene expression to reference genomes, some studies integrate RNA-seq analyses with targeted metabolomics to provide deeper insights into functional dynamics. For example, Bodinaku et al. (80) examined shifts in the metabolic processes of *Penicillium* strains on cheese rinds during domestication and revealed strain-specific metabolic down-regulations at the transcriptional level (80). Similarly, Vicente et al. (81) combined metabolomics and transcriptomics to show how within-species transcriptomic diversity in *Lachancea thermotolerans* drives acidification activity in wine-making (82).

However, metatranscriptomics still faces several challenges, starting with sample collection and RNA preservation, which are critical due to the short half-life of mRNA transcripts (77). The presence of host RNA contamination (e.g., from food substrate matrices) and the requirement for efficient rRNA depletion add further complexity (83). Additionally, the use of short-read sequencing methods for mRNA libraries limits the resolution of complex features such as RNA isoforms, operon structures, and full-length transcripts, which may be critical for achieving strain-level insights (77, 84) and can distort the representation of certain microorganisms and their strain-specific, transient transcriptional patterns. These technical hurdles, coupled with higher costs, lower scalability compared to DNA sequencing protocols, and the immaturity of currently available statistical analysis models (85), still restrict the widespread adoption of metatranscriptomics in large-scale food microbiome surveys.

While transcript levels can be used as a proxy to understand functional responses of communities, they do not always correlate with actual functional changes (86). A more robust indicator is metaproteomics, which confirms the presence of functional proteins, hence providing a direct link to microbial activity, enzyme-related traits, protein-protein interactions, and the discovery of relevant proteins (87, 88). Currently, the lower database coverage compared to genomic databases and the complexity of non-microbial proteins in substrate matrices limit the resolution of metaproteomics in foods (59). Nonetheless, future advances in high-throughput metaproteomics methods may enhance the study of strain-specific enzyme abundance and functional traits, directly impacting the diverse flavors and nutritional values (89–91).

### Metabolomics is a key tool for understanding microbes in their ecosystem context

Microbes are influenced by the chemical environment surrounding them, including molecules, salts, and other compounds that affect their behavior, metabolism, and interactions. The chemical milieu shapes within-species diversity and is in turn influenced by the strain-specific functional capabilities of microbes. While metagenomics and metatranscriptomics provide insights into the potential metabolic capabilities of a strain, metabolomics—studying small molecules produced by metabolic processes—offers a direct representation of the phenotype. It is therefore an important tool to understand microbial behavior within the respective ecosystems (92).

Metabolomics is useful to explore the dynamics within microbial communities and interspecies interactions. For example, Blasche et al. (93) show how metabolic cooperation leads to stable coexistence of multiple strains within microbial consortia in milk kefir (93). Moreover, metabolomics can be employed to elucidate the specific function of individual strains in communities. This was shown by Rappaport et al. (4), which combined metabolomics, specifically analysis of VOCs, and genomic characterization of synthetic communities with varying strain composition to demonstrate that the function, i.e. aroma formation, of the entire community can vary depending on within-species genetic differences (4). Inversely to focusing on desired functions, metabolomics is applied to reveal which community members contribute to food defects or spoilage to increase food safety. An example thereof is the study by Liu et al. (94) which applied metabolomics coupled with NGS-guided screening of strains to reduce the formation of biogenic amines in rice wine (94). Alternatively, metabolomics is used to reveal how the chemical composition shapes microbial communities. This was done by Yap et al. (95), which connected environmental and climatic conditions with chemical profiles and metagenomic microbiome analysis in raw milk to reveal the importance of environmental context in shaping the strain composition (95). These studies collectively illustrate the power of metabolomics in providing insights into microbial community dynamics and function, as well as ecosystem interactions. In particular, the integration of metabolomics with genetic and ecological data is central in food systems for revealing strain-specific metabolite profiles, their roles in community metabolism, and adaptations to environmental changes leading to strain stratification (90).

### Understanding how viruses influence within-species diversity and the implications for microbiome function and stability

Microbial systems are a rich reservoir for the development and propagation of diverse microorganism-infecting viruses (96). In bacteria, the susceptibility to viruses (bacteriophages) can vary dramatically within a species, and the presence thereof shapes communities by reducing the abundance of certain members or fostering mutualistic relationships through prophage domestication, where a bacteriophage integrates into the host genome (97, 98). Bacteriophages can also protect bacteria via superinfection exclusion, by reducing the reinfection rates of the same or related bacteriophages (99). The selective pressure imposed by constant bacteriophage predation can lead to diversification of and within species through HGT and induce drastic community shifts. This poses a risk to the reliability of industrial starter cultures, e.g., the viral infection of dairy lactic acid bacteria starter cultures is a common cause for failed fermentations (98). Yet, viral selective pressure can also maintain low within-species diversity and act as natural biocontrol, contributing to long-term community stability and preventing the establishment of competitor strains (100, 101). The role of viruses in nutrient redistribution, by releasing organic matter through host cell lysis, is termed “viral shunt” and has been suggested to facilitate succession of microbial populations in food fermentations (102). Fungi, like bacteria, are also susceptible to viral infections (mycoviruses), as demonstrated in *Saccharomyces cerevisiae*, which can be chronically infected with the mycovirus L-A (103). Additionally, certain viruses can occasionally convert specific yeast strains into toxin-producing “killer strains” (104).

Investigating viruses and within-species diversity requires examining bacteriophage-bacterial or mycovirus-fungal coevolution as well as associated clustered regularly interspaced short palindromic repeats (CRISPR) resistance arrays with metagenomics or viromics, along with targeted phage isolation and *in vitro* characterization (105, 106). An example combining all of these approaches is Somerville et al. (101) who explored within-species bacterial diversity and bacteriophage resistance in cheese starter cultures to reveal interspecies functional redundancy alongside significant strain-level variation in virus resistance (101). Furthermore, analyzing CRISPR arrays or isolated viruses from, e.g., production sites may elucidate phylogenies in fermentations down to strain level and deepen our understanding of strain-level dynamics in fermentation processes in the future (102, 107).

## CONCLUSION AND OUTLOOK

Characterizing the phenotypes and traits of individual microbial strains has long been recognized as essential, as even closely related microorganisms within the same species can exhibit markedly different functions. Strain variation is particularly evident in food systems, affecting characteristics such as flavor, texture, and safety (9). Moreover, microbial biodiversity is a critical resource for food innovation and human health (108). Unlocking this potential requires a comprehensive understanding of within-species diversity and its implications. Here, we outlined a roadmap integrating emerging technologies with innovative methodologies and present a multifaceted outlook on future technological developments and the opportunities they may offer.

High-resolution community analyses, such as long-read marker-gene sequencing, shotgun metagenomics, or scDNA-seq, are becoming more accessible with advancements in technology and computational power (109). In particular, advances in scDNA-seq hold the potential to revolutionize the study of within-species diversity (65) and could be particularly relevant in assessing genetic heterogeneities within communities, to study, for example, the spread of antibiotic resistances and mechanisms of persistence in spoilage organisms, or the genetic variations in and the dispersion thereof in fermented foods. High-resolution sequencing technologies will be essential to the discovery of novel strains, as well as elucidating evolution and ecology thereof. Therefore, despite remaining challenges, we anticipate these technologies to become more commonly used to investigate fundamental questions in microbiology as well as in practical applications such as tracing contamination routes in food production and authenticating food products (109, 110). Shotgun metagenomics further enables the exploration of the functional potential of individual strains or entire microbial communities and could super-charge strain isolation through culturomics, enabling more precise strain selection for targeted food applications (111) and potentially paving the way for personalized dietary recommendations based on microbial profiles (112, 113).

Advances in bioinformatics and machine learning tools will facilitate predictive modeling to assess food quality and safety based on metagenomic profiles (114, 115), more accurate mapping of transcripts or proteins to strain level-resolved reference databases (75), and prediction of strain-specific functional responses from multi-omics data (116, 117). These tools will aid in distinguishing functional differences between strains, assessing their impact on food quality, identifying key strains and functions responsible for desired flavors and nutritional values, and supporting targeted interventions to enhance quality and safety.

Although metaproteomics is still a nascent field, it is rapidly evolving and promises insights into protein-protein interactions within microbial communities (118). Future advances in protein identification and quantification in complex food matrices will enhance the ability to detect low-abundance proteins and strain-specific enzymes. Ultimately, longitudinal studies could further reveal temporal changes in gene expression and protein abundance, shedding light on community dynamics and strain-specific functional adaptations throughout, for example, the fermentation process.

Similarly, future advancements in metabolomics technologies will enhance the detection of low-abundance metabolites and improve spatial resolution, allowing for the capture of metabolite gradients within microbial communities, which could facilitate the discovery and implementation of distinct biomarkers for food quality and safety (119). Real-time metabolite profiling may also enable the tracking of dynamic changes in microbial ecosystems, offering a future spatio-temporal understanding of communities and their strain-specific contributions. Further multi-omics integrations can link metabolomics to genetic potential, correlate metabolite production with gene expression, and connect metabolomics to metaproteomics to explore the relationship between enzymes and metabolites. Analyzing these multi-omics layers will require advancements in statistical and bioinformatics techniques for interpreting cross-talk between data layers. Supported by artificial intelligence, such methods could enable the prediction of metabolic interactions and community behavior based on genomic or environmental data, thereby identifying key metabolites that drive community dynamics and functionality (120, 121).

Viruses are a frequently overlooked yet crucial component of microbial ecosystems, shaping community composition through strain-specific susceptibilities. Future technological advancements aiming at high-resolution analysis of viral population dynamics within microbiomes will improve the detection, characterization, and isolation of viruses associated with microbial communities. These advancements will also facilitate the screening of interactions with specific strains and the identification of potential biocontrol applications (122). Integration of such strain-specific data will facilitate the modeling of virus-microbe dynamics along with their impact on microbial community stability and (co-)evolution (123). Further integration with metatranscriptomics and metabolomics could help understand microbial gene expression in response to viruses and direct impact on community function. Additionally, CRISPR array analysis has the potential to uncover viral histories and strain-specific resistance mechanisms (102, 107), supporting targeted biocontrol strategies applicable in industrial scales. Collectively, these developments promise to deepen our understanding of the fundamental role of viruses in microbial ecosystems, aiding the design of resilient starter cultures, phage control strategies, and innovative biotechnological applications such as virus-induced diversification of fermented food microbiomes (124).

While the described omics technologies support revealing the origins and implications of within-species diversity, the isolation and *in vitro* testing of strains remains a central technique in microbiology. Physical strain isolates are key to obtaining high-quality assembled genomes and enabling experimental manipulation (125), which can reveal true causal connections and allow the study of interactions within communities (2). Ultimately, to gain a deeper understanding of how microbial communities evolve and to generate practical insights for food producers, experimental manipulations in both synthetic environments and real-world food production settings are essential. These experimental approaches enable the exploration of strain behaviors and interactions under more complex conditions, complementing the insights gained from omics tools.

Notably, these various experimental approaches need to be tailored to the various microbial groups present in fermented foods, such as bacteria, yeast, and molds. Given that the definition of “strain” often varies across research fields, future research must account for these differences and tackle specific challenges, such as capturing fungal proteins and addressing discrepancies in database coverage between kingdoms. Integrating these approaches will ultimately be crucial for comprehensively understanding within-species diversity and its role in inter-kingdom interactions within food systems.

In conclusion, this review highlights the importance of investigating within-species diversity in microbial communities of food systems, as it is necessary for driving deeper insight into the functional roles and interactions of microorganisms within these ecosystems. The integrated application of novel or existing techniques, including paired integration of cultivation-based techniques and omics technologies, can radically advance our fundamental understanding of microbiomes, while driving significant innovations in food production to enhance quality, safety, and production efficiency.
